# Rates of childbirth in female cancer survivors: A population-based study in Taiwan

**DOI:** 10.1371/journal.pone.0358466

**Published:** 2026-09-11

**Authors:** Ya-Ling Hsieh, Chia-Jung Chiang, Tsung Yu

**Affiliations:** 1 Nursing Department, Chi Mei Medical Center, Tainan, Taiwan; 2 Department of Public Health, College of Medicine, National Cheng Kung University, Tainan, Taiwan; 3 Department of Obstetrics and Gynecology, Kaohsiung Veterans General Hospital Tainan Branch, Tainan, Taiwan; E-Da Cancer Hospital, TAIWAN

## Abstract

**Background:**

As cancer survival improves, increasing numbers of women diagnosed during their reproductive years face potential fertility impairment. However, population-based evidence on post-cancer childbearing remains limited in Asian settings.

**Methods:**

We conducted a nationwide cohort study in Taiwan by linking the Cancer Registry, Birth Reporting Registry, and Death Registry. A total of 72,929 females diagnosed with cancer at age ≤ 39 years were followed to identify subsequent livebirths. The cumulative incidence of first post-diagnosis livebirth was estimated using competing-risk methods. Predictors were evaluated using cause-specific Cox models. Standardized birth ratios (SBRs) were calculated by comparing observed births with expected births in the general population, standardized by age and calendar year.

**Results:**

Overall, 9.8% of female cancer survivors had at least one livebirth after diagnosis. The 5-, 10-, and 15-year cumulative incidences were 6.4%, 10.7%, and 13.9%, respectively. Compared with the general population, survivors had approximately half the expected number of births (SBR = 0.50; 95% CI, 0.49–0.51). Substantial heterogeneity was observed across cancer types: childbirth was most preserved among survivors of thyroid and skin cancers, whereas those with cervical, uterine, breast, leukemia, and central nervous system cancers had markedly reduced childbirth. Older age at diagnosis and receipt of chemotherapy were strongly associated with lower likelihood of childbirth.

**Conclusions:**

In this large, nationwide study, female cancer survivors in Taiwan experienced substantially reduced childbirth rates, with pronounced variation by cancer type, age at diagnosis, and treatment. These findings provide robust population-level evidence from an Asian context and highlight the need for early fertility counseling and improved access to oncofertility care.

## Introduction

Cancer can be diagnosed during childhood and the reproductive years. In 2022, a total of 62,994 new cancer cases were diagnosed among females in Taiwan. Of these, 170 occurred in children aged 0–14 years and 4,036 in adolescents and young adults aged 15–39 years, representing 6.7% of all new diagnoses [1]. With continued advances in cancer detection and treatment, survival has markedly improved in Taiwan: the 5-year survival rate for females increased from 53.2% among those diagnosed in 2002 to 65.5% among those diagnosed in 2018 [2]. As more patients live beyond their diagnosis, growing numbers of childhood, adolescent, and young adult cancer survivors face long-term and late effects of the cancer and treatment, including chronic health conditions, secondary malignancies, impaired fertility, psychosocial challenges, and financial toxicity [3–5].

Fertility is a central concern for individuals diagnosed with cancer during their reproductive years. Many cancer-related treatments—including chemotherapy, radiotherapy, and surgical procedures—can substantially compromise reproductive function. Accordingly, early integration of fertility preservation into treatment planning and survivorship care is critical [6]. The American Society of Clinical Oncology (ASCO) 2025 guideline offers comprehensive, evidence-based recommendations for both adult and pediatric populations. It strongly emphasizes the need for clinicians to discuss potential infertility risks before treatment begins, provide timely referrals to reproductive specialists, and present age- and sex-appropriate fertility preservation options [7]. Although these recommendations underscore the importance of preserving reproductive potential as a fundamental component of cancer care for young patients, evidence on long-term reproductive outcomes across different populations is still needed.

For example, population‐based studies from Western countries consistently demonstrate reduced childbearing among cancer survivors. A Swedish registry study reported a 27% lower likelihood of childbirth among survivors compared with the general population [8], while data from Scotland showed a 38% reduction in the probability of subsequent pregnancy [9]. In the United States, a study of female adolescent and young adult survivors in North Carolina found that their birth rate was only 55% of that of age- and race-matched women without cancer [10]. But comparable data from Asian populations, including Taiwan, remain limited.

In Taiwan, to strengthen reproductive support for cancer patients and reduce financial barriers, the Ministry of Health and Welfare launched a national pilot fertility preservation subsidy program in September 2025 [11]. Previously, subsidies for fertility preservation related to iatrogenic infertility were administered by local governments, resulting in substantial regional disparities in access. Under the new policy, for patients aged 18–40 years with breast cancer or hematologic malignancies, the national program covers oocyte cryopreservation for female patients and sperm cryopreservation for male patients before the initiation of gonadotoxic treatment. By supporting pre-treatment gamete cryopreservation, the program aims to preserve future reproductive potential before irreversible impairment of ovarian or testicular function occurs. As this national initiative evolves, robust population-based evidence on reproductive outcomes after cancer will be essential to guide policy refinement, expansion of eligibility criteria, and equitable allocation of healthcare resources.

Given the absence of population-based data on childbirth after cancer in Taiwan, we conducted a nationwide, registry-based cohort study to examine the cumulative incidence of livebirth after a cancer diagnosis at age ≤ 39 years among Taiwanese females, and to assess variation by cancer type, age at diagnosis, calendar period, level of urbanization, and cancer treatment.

## Methods

### Data source

Study data were obtained on September 1, 2025 through linkage of multiple nationwide registries in Taiwan, where each resident is assigned a unique personal identification number. Information on cancer diagnosis (type and date), residential location, and first-course treatments was derived from the Taiwan Cancer Registry (from 1979 to 2022), a population-based system to record all newly diagnosed cancer cases [12]. Data on livebirths were obtained from the Birth Reporting Registry (from 2001 to 2023), which requires the birth attendant to report birth information to authorized health agencies within seven days of delivery [13]. Mortality data were retrieved from the Taiwan Death Registry (up to 2023).

From the Taiwan Cancer Registry, we identified all first invasive cancer diagnoses among women aged ≤39 years. Cancer types were classified into mutually exclusive categories according to the ICD-O-3/WHO 2008 definitions, with cancer diagnoses across different calendar periods harmonized to this common classification system: oral cavity and pharynx; nasopharynx; digestive system; colon and rectum; liver and intrahepatic bile duct; respiratory system; connective and soft tissue; skin; breast; cervix; uterus; ovary; urinary and other genital system; eye, brain and other nervous system; thyroid; lymphoma; leukemia; and other. Age at cancer diagnosis was grouped into six categories: 0–14, 15–19, 20–24, 25–29, 30–34, and 35–39 years. Calendar year of cancer onset was categorized into ≤2000, 2001–2004, 2005–2009, 2010–2014, 2015–2019, and 2020–2022. Level of urbanization was classified as rural, satellite, or urban, based on the report from the Center for Survey Research, Research Center for Humanities and Social Sciences, Academia Sinica [14]. Cancer treatment was categorized into four mutually exclusive groups based on a clinical hierarchy: (1) chemotherapy (with or without surgery/radiation); (2) radiation, no chemotherapy (with or without surgery); (3) surgery only; and (4) other (including unknown or unspecified treatments).

This study protocol was approved by the Institutional Review Board of National Cheng Kung University Hospital, Tainan, Taiwan. Datasets were obtained from the Health and Welfare Data Science Center, Ministry of Health and Welfare in Taiwan.

### Subsequent first childbirth for women with cancer

Follow-up began on the date of cancer diagnosis. For individuals diagnosed before age 15 years, follow-up commenced at age 15. Women were followed until the earliest of the following events: first post-diagnosis livebirth, age 49 years, or the end of 2023. The earliest eligible patients in our cohort were those diagnosed during infancy in 1986, who subsequently survived and entered their reproductive age within our 2001–2023 tracking window; conversely, the latest eligible patients were those diagnosed in 2022 and followed through 2023. We excluded women with less than one year of follow-up; therefore, births occurring within one year after cancer diagnosis were not included. Thus, all included women conceived at least three months after their cancer diagnosis. This specific exclusion criteria was established to ensure that all recorded livebirths represented new conceptions initiated strictly during the post-diagnostic survivorship period [8].

We estimated the 5-, 10-, and 15-year cumulative incidence of first livebirth after cancer using PROC LIFETEST in SAS, treating death as a competing event [15]. To evaluate predictors of first livebirth, we fit cause-specific Cox models using PROC PHREG to obtain hazard ratios (HRs) and 95% confidence intervals (CIs) [16]. Predictors included cancer type, age at diagnosis, period of cancer onset, level of urbanization, and cancer treatment. Multivariable models were performed that adjusted for cancer type, age at diagnosis, and period of cancer onset. We did not include cancer treatment in the models since it is a mediator rather than a confounding factor in the relationship between cancer diagnosis and subsequent childbirth. Level of urbanization was also not included due to its non-significant impact in the univariate model.

To evaluate chronological or ordinal linear trends across grouped categories (such as age at diagnosis and period of cancer onset), the categorical variables were converted into ordinal scores by assigning sequential integers (e.g., 1, 2, 3, 4...) and the statistical significance of the linear trend was assessed. Proportional hazards assumptions were assessed by inspecting log(–log[survival]) versus log(time) plots.

### Standardized birth ratio (SBR) for women with cancer

To compare the birth rate among women diagnosed with cancer at age ≤ 39 years with that of the general female population in Taiwan, we applied indirect standardization to estimate the SBR [9]. The SBR was defined as the ratio of observed livebirths among women with cancer to the expected number of livebirths in the reference population, standardized by age and calendar year. The SBR approach was chosen because individual-level data for a matched non-cancer reference cohort were unavailable to our research team due to data acquisition and administrative restrictions. By standardizing for both exact age and specific calendar years, this approach effectively controls for the substantial macro-level temporal declines in fertility rates observed in Taiwan over recent decades.

For women with cancer, person-years at risk for childbirth were accrued beginning one year after the cancer diagnosis or from age 15 years (whichever occurred later) until age 49 years or death. Background birth rates were calculated from national data on livebirths and mid-year female population counts, stratified by 5-year age and calendar year groups. Expected births were obtained by multiplying the stratum-specific background birth rate by the corresponding person-years at risk, and then summing across all strata. The SBR was calculated by dividing the total number of observed livebirths by the total expected number. SBRs were estimated separately by cancer type, age at diagnosis, period of cancer onset, level of urbanization, and cancer treatment.

All data management and analyses were performed using SAS version 9.4 (SAS Institute, Cary, NC, USA).

## Results

### Characteristics of women with cancer

We included 72,929 female cancer survivors in our analysis (Table 1). The five most common cancer types were breast (29.5%), thyroid (18.5%), ovarian (6.0%), cervical (5.9%), and colorectal cancer (5.4%). Nearly half of the women (46.6%) were diagnosed between ages 35 and 39 years. Most diagnoses occurred between 2001 and 2022, while only 2.4% were diagnosed in or before 2000. It was because electronic livebirth events could only be captured from 2001 onward, patients diagnosed in 2000 or earlier were only captured in this cohort if they were diagnosed at a very young age (e.g., childhood or adolescence) and survived to potentially achieve a livebirth within our 2001–2023 tracking window. Overall, 43.6% of women resided in urban areas, and 45.5% received chemotherapy.

**Table 1:** Characteristics of the study cohort of Taiwanese women with cancer onset at age ≤ 39 years.

	Women with cancer	
	Number	%
Total	72929	100
Type of cancer		
Breast	21489	29.5
Thyroid	13519	18.5
Ovary	4380	6.0
Cervix	4270	5.9
Colon and rectum	3948	5.4
Leukemia	3524	4.8
Lymphoma	3455	4.7
Uterus	3294	4.5
Respiratory system	2261	3.1
Eye, brain and other nervous system	2003	2.8
Digestive system^a^	1941	2.7
Connective and soft tissue	1576	2.2
Nasopharynx	1572	2.2
Skin	1337	1.8
Oral cavity and pharynx^b^	1279	1.8
Urinary and other genital system^c^	1265	1.7
Liver and intrahepatic bile duct	943	1.3
Other^d^	873	1.2
Age at cancer diagnosis (years)		
0-14	3942	5.4
15-19	2389	3.3
20-24	4552	6.2
25-29	9394	12.9
30-34	18636	25.6
35-39	34016	46.6
Period of cancer onset		
≤2000	1747	2.4
2001-2004	11103	15.2
2005-2009	14693	20.2
2010-2014	16264	22.3
2015-2019	18186	24.9
2020-2022	10936	15.0
Level of urbanization		
Rural	12468	17.1
Satellite	28398	38.9
Urban	31791	43.6
Treatment of cancer		
Surgery only	17417	23.9
Radiation, no chemotherapy	11942	16.4
Chemotherapy	33210	45.5
Other	10360	14.2

^a^Not including “colon and rectum” and “liver and intrahepatic bile duct”

^b^Not including “nasopharynx”

^c^Not including “ovary”, “cervix” and “uterus”

^d^Including “other endocrine system” and “miscellaneous”.

### First ever childbirth following cancer diagnosis

Among the 72,929 cancer survivors, 7,179 (9.8%) had at least one livebirth occurring one year or more after their cancer diagnosis. The median follow-up duration was 7.6 years (interquartile range [IQR], 3.6–11.9 years). Among the 7,179 women who had post-diagnosis livebirths, the median time from diagnosis to first livebirth was 4.3 years (IQR, 2.6–7.3 years). Overall, the 5-, 10-, and 15-year cumulative incidence of post-diagnosis livebirth was 6.4%, 10.7%, and 13.9%, respectively (Table 2).

**Table 2:** Distribution of events, survival time and estimates of cumulative incidence of livebirth following cancer.

	Event of first livebirth		Event of death		Survival time (person-years)	Cumulative incidence of livebirth following cancer (%)		
	Number	%	Number	%		5-year	10-year	15-year
Total	7179	9.8	10920	15.0	596605	6.4	10.7	13.9
Type of cancer								
Breast	1138	5.3	3577	16.7	174216	3.4	6.5	7.2
Thyroid	2362	17.5	122	0.9	109534	12.3	20.3	25.9
Ovary	605	13.8	594	13.6	37166	8.5	14.0	18.5
Cervix	183	4.3	688	16.1	38218	3.8	4.6	4.9
Colon and rectum	277	7.0	1178	29.8	28302	5.1	8.0	8.7
Leukemia	280	8.0	584	16.6	32477	3.0	6.2	11.3
Lymphoma	524	15.2	325	9.4	30052	8.1	16.0	21.6
Uterus	149	4.5	271	8.2	26573	4.2	5.4	5.4
Respiratory system	152	6.7	809	35.8	11339	5.9	9.0	10.5
Eye, brain and other nervous system	150	7.5	571	28.5	18714	2.4	5.8	9.8
Digestive system	163	8.4	609	31.4	13711	5.8	9.3	11.4
Connective and soft tissue	212	13.5	380	24.1	13092	6.1	11.8	17.9
Nasopharynx	173	11.0	297	18.9	14100	7.6	11.2	13.3
Skin	268	20.0	90	6.7	12977	11.4	18.8	25.3
Oral cavity and pharynx	216	16.9	163	12.7	10785	10.8	16.8	23.3
Urinary and other genital system	173	13.7	188	14.9	10782	9.0	14.1	17.4
Liver and intrahepatic bile duct	87	9.2	329	34.9	7262	6.1	9.3	10.9
Other	67	7.7	145	16.6	7304	4.5	8.0	10.7
Age at cancer diagnosis (years)								
0-14	351	8.9	192	4.9	47191	1.0	4.1	10.8
15-19	442	18.5	352	14.7	22234	3.0	11.7	27.6
20-24	1124	24.7	586	12.9	38534	9.6	25.1	36.7
25-29	2211	23.5	1331	14.2	75050	15.9	27.8	30.4
30-34	2239	12.0	2908	15.6	156527	9.8	14.0	14.6
35-39	812	2.4	5551	16.3	257069	2.3	2.7	Not estimable
Period of cancer onset								
≤2000	197	11.3	55	3.2	28660	1.1	3.6	8.7
2001-2004	1290	11.6	2592	23.4	129648	4.9	8.6	11.9
2005-2009	1846	12.6	3040	20.7	159774	6.0	10.6	13.4
2010-2014	1995	12.3	2777	17.1	147539	7.1	11.8	Not estimable
2015-2019	1560	8.6	1996	11.0	104657	7.2	Not estimable	Not estimable
2020-2022	291	2.7	460	4.2	26328	Not estimable	Not estimable	Not estimable
Level of urbanization								
Rural	1221	9.8	2024	16.2	103764	5.9	10.6	13.5
Satellite	2805	9.9	4231	14.9	229152	6.3	10.8	14.1
Urban	3123	9.8	4625	14.6	261129	6.5	10.6	13.7
Treatment of cancer								
Surgery only	2356	13.5	1209	6.9	155694	9.1	14.1	18.0
Radiation, no chemotherapy	1527	12.8	722	6.1	93206	8.8	15.4	19.9
Chemotherapy	2057	6.2	7785	23.4	259572	3.5	6.7	9.1
Other	1239	12.0	1204	11.6	88133	8.4	12.7	15.7

The likelihood of post-diagnosis childbirth varied substantially across cancer types. The highest 15-year cumulative incidences were observed among survivors of thyroid (25.9%), skin (25.3%), oral cavity and pharyngeal cancers (23.3%), and lymphoma (21.6%), whereas the lowest incidences occurred among survivors of cervical (4.9%) and uterine cancers (5.4%). Additional cumulative incidence estimates stratified by age at diagnosis, period of cancer onset, level of urbanization, and treatment modality are presented in Table 2.

Multivariable cause-specific Cox models demonstrated persistent heterogeneity in childbirth outcomes by cancer type (Table 3). Using breast cancer as the reference, significantly higher likelihood of subsequent childbirth was observed among survivors of skin cancer (adjusted HR [aHR] = 2.17; 95% CI, 1.90–2.49), thyroid cancer (aHR = 2.08; 1.94–2.24), urinary and other genital cancers (aHR = 2.06; 1.75–2.42), and oral cavity and pharyngeal cancers (aHR = 2.03; 1.75–2.35). In contrast, markedly reduced likelihood was seen among survivors of cervical (aHR = 0.71; 0.61–0.83) and uterine cancers (aHR = 0.72; 0.61–0.85), consistent with the potential detrimental effects of treatment involving reproductive organs.

**Table 3:** Hazard ratio (HR) and 95% confidence interval (CI) for subsequent first childbirth in women with cancer onset at age ≤ 39 years.

	Unadjusted HR	95% CI	Adjusted HR^a^	95% CI
Type of cancer				
Breast	1.00	–	1.00	–
Thyroid	3.35	3.12, 3.60	2.08	1.94, 2.24
Ovary	2.56	2.32, 2.83	1.57	1.42, 1.74
Cervix	0.76	0.65, 0.89	0.71	0.61, 0.83
Colon and rectum	1.53	1.34, 1.74	1.31	1.15, 1.49
Leukemia	1.39	1.22, 1.58	0.96	0.84, 1.11
Lymphoma	2.75	2.48, 3.05	1.51	1.36, 1.68
Uterus	0.87	0.73, 1.03	0.72	0.61, 0.85
Respiratory system	2.13	1.80, 2.52	1.87	1.58, 2.22
Eye, brain and other nervous system	1.30	1.10, 1.55	0.93	0.78, 1.10
Digestive system	1.90	1.61, 2.24	1.44	1.22, 1.70
Connective and soft tissue	2.59	2.24, 3.00	1.59	1.36, 1.84
Nasopharynx	1.92	1.64, 2.26	1.54	1.31, 1.81
Skin	3.29	2.88, 3.75	2.17	1.90, 2.49
Oral cavity and pharynx	3.15	2.73, 3.65	2.03	1.75, 2.35
Urinary and other genital system	2.54	2.16, 2.98	2.06	1.75, 2.42
Liver and intrahepatic bile duct	1.92	1.54, 2.39	1.54	1.24, 1.92
Other	1.47	1.15, 1.88	1.10	0.86, 1.41
Age at cancer diagnosis (years)				
0-14	1.00	–	1.00	–
15-19	2.54	2.21, 2.92	1.95	1.62, 2.35
20-24	3.72	3.30, 4.19	2.82	2.37, 3.35
25-29	3.81	3.40, 4.26	3.14	2.66, 3.72
30-34	1.82	1.63, 2.04	1.64	1.38, 1.94
35-39	0.38	0.33, 0.43	0.36	0.30, 0.43
Period of cancer onset				
≤2000	1.00	–	1.00	–
2001-2004	1.42	1.23, 1.66	0.85	0.68, 1.06
2005-2009	1.64	1.41, 1.90	0.97	0.78, 1.21
2010-2014	1.85	1.60, 2.15	1.11	0.89, 1.38
2015-2019	1.88	1.62, 2.19	1.18	0.95, 1.47
2020-2022	1.92	1.59, 2.31	1.18	0.92, 1.51
*p for trend after 2001*	*<0.001*		*<0.001*	
Level of urbanization				
Rural	1.00	–		
Satellite	1.04	0.97, 1.11		
Urban	1.01	0.95, 1.08		
Treatment of cancer				
Surgery only	1.00			
Radiation, no chemotherapy	1.06	0.99, 1.13		
Chemotherapy	0.52	0.49, 0.55		
Other	0.93	0.87, 1.00		

^a^Adjusted for type of cancer, age at cancer diagnosis and period of cancer onset.

Reproductive potential also varied by age at diagnosis. Compared with those diagnosed at ages 0–14 years, the likelihood of childbirth was higher for women diagnosed at 15–19 (aHR = 1.95; 1.62–2.35), 20–24 (aHR = 2.82; 2.37–3.35), 25–29 (aHR = 3.14; 2.66–3.72), and 30–34 years (aHR = 1.64; 1.38–1.94), whereas women diagnosed at 35–39 years had substantially lower likelihood (aHR = 0.36; 0.30–0.43). With respect to calendar period, crude estimates suggested increasing cumulative incidence in more recent diagnostic years (p-trend <0.001), and a significant trend persisted after adjustment (p < 0.001). Level of urbanization was not significantly associated with childbirth. Regarding treatment, compared with surgery alone, chemotherapy was associated with a 48% reduction in subsequent childbirth (unadjusted HR = 0.52; 0.49–0.55).

### All childbirths following cancer diagnosis

As shown in Table 4, women diagnosed with cancer at age ≤ 39 years had substantially fewer livebirths than expected based on the general population in Taiwan, with an overall SBR of 0.50 (95% CI, 0.49–0.51), indicating a 50% reduction in post-diagnosis childbearing. Birth outcomes varied considerably by cancer type. Survivors of skin cancer (SBR = 0.77; 95% CI, 0.69–0.84) and thyroid cancer (SBR = 0.73; 95% CI, 0.70–0.75) had birth rates closest to the background population, whereas those with cancers of the cervix (SBR = 0.26; 95% CI, 0.23–0.29), uterus (SBR = 0.27; 95% CI, 0.24–0.31), and breast (SBR = 0.33; 95% CI, 0.31–0.35) were among the least likely to give birth. Similarly low ratios were seen for leukemia (SBR = 0.35; 95% CI, 0.32–0.39) and tumors of the eye, brain, and other nervous system (SBR = 0.31; 95% CI, 0.27–0.35).

**Table 4:** Standardized birth ratio (SBR) for childbirth in women with cancer onset at age ≤ 39 years.

	Observed livebirths	Expected livebirths	SBR^a^	95% CI
Total	10304	20725	0.50	0.49, 0.51
Type of cancer				
Breast	1563	4733	0.33	0.31, 0.35
Thyroid	3372	4630	0.73	0.70, 0.75
Ovary	930	1543	0.60	0.56, 0.64
Cervix	261	1003	0.26	0.23, 0.29
Colon and rectum	385	940	0.41	0.37, 0.45
Leukemia	419	1188	0.35	0.32, 0.39
Lymphoma	766	1312	0.58	0.54, 0.63
Uterus	227	830	0.27	0.24, 0.31
Respiratory system	192	435	0.44	0.38, 0.51
Eye, brain and other nervous system	227	732	0.31	0.27, 0.35
Digestive system	232	501	0.46	0.41, 0.52
Connective and soft tissue	327	547	0.60	0.54, 0.66
Nasopharynx	247	465	0.53	0.47, 0.60
Skin	407	531	0.77	0.69, 0.84
Oral cavity and pharynx	289	435	0.66	0.59, 0.74
Urinary and other genital system	248	381	0.65	0.57, 0.73
Liver and intrahepatic bile duct	115	255	0.45	0.37, 0.54
Other	97	267	0.36	0.29, 0.44
Age at cancer diagnosis (years)				
0-14	541	1481	0.37	0.34, 0.40
15-19	726	908	0.80	0.74, 0.86
20-24	1810	2497	0.72	0.69, 0.76
25-29	3310	5295	0.63	0.60, 0.65
30-34	2957	6566	0.45	0.43, 0.47
35-39	960	3978	0.24	0.23, 0.26
Period of cancer onset				
≤2000	327	1078	0.30	0.27, 0.34
2001-2004	2003	3780	0.53	0.51, 0.55
2005-2009	2795	5037	0.55	0.53, 0.58
2010-2014	2855	5283	0.54	0.52, 0.56
2015-2019	1981	4255	0.47	0.45, 0.49
2020-2022	343	1293	0.27	0.24, 0.29
Level of urbanization				
Rural	1834	3690	0.50	0.47, 0.52
Satellite	4046	8052	0.50	0.49, 0.52
Urban	4377	8888	0.49	0.48, 0.51
Treatment of cancer				
Surgery only	3467	5476	0.63	0.61, 0.65
Radiation, no chemotherapy	2166	3558	0.61	0.58, 0.63
Chemotherapy	2923	8340	0.35	0.34, 0.36
Other	1748	3350	0.52	0.50, 0.55

^a^Standardized for age and calendar year.

Clear age-related gradients were observed. Survivors diagnosed during adolescence (15–19 years) had the highest SBR (0.80; 95% CI, 0.74–0.86), followed by those diagnosed at 20–24 years (0.72; 95% CI, 0.69–0.76). SBR declined steadily with older age at diagnosis and was lowest among women diagnosed at 35–39 years (0.24; 95% CI, 0.23–0.26). Birth rates also differed by calendar period, with higher ratios among women diagnosed in 2005–2009 (0.55; 95% CI, 0.53–0.58) and 2010–2014 (0.54; 95% CI, 0.52–0.56), followed by a decline among more recently diagnosed women, likely reflecting shorter follow-up durations.

Across levels of urbanization, SBRs were comparable (0.49–0.50). When stratified by treatment, the likelihood of childbirth was highest among women treated with surgery only (SBR = 0.63; 95% CI, 0.61–0.65) and lowest among those who received chemotherapy (SBR = 0.35; 95% CI, 0.34–0.36).

## Discussion

In this nationwide cohort of Taiwanese women diagnosed with cancer at age ≤ 39 years, only about one in ten had a livebirth after diagnosis, and overall birth rates were roughly 50% lower than expected compared with age- and calendar year–matched women in the general population. These findings highlight the substantial impact of cancer on subsequent childbearing in Taiwan. Considerable heterogeneity was observed: the likelihood of livebirth was highest among survivors of skin and thyroid cancers, whereas women with gynecological cancers (cervical and uterine), leukemia, breast cancer, and tumors of the eye, brain, and nervous system experienced the largest reductions. Birth rates also declined with increasing age at diagnosis and were especially low among women treated with chemotherapy.

There is a notable lack of studies from Asian populations examining childbirth after cancer. Our findings are broadly consistent with population-based evidence from Europe and North America [8–10,17], which typically report a 20%–50% reduction in pregnancy or childbirth after cancer and substantial variation by cancer type. Prior research has also identified particularly low post-diagnosis childbirth among survivors of leukemia, central nervous system (CNS) tumors, breast cancer, and gynecological malignancies, with comparatively preserved likelihood of childbirth after melanoma and thyroid cancer [8–10,17]. Several studies further emphasize the influence of parity at diagnosis: nulliparous survivors often have pregnancy rates more comparable to those of the general population, whereas parous survivors show more pronounced declines [8,18].

The heterogeneity observed across cancer types likely reflects differences in both disease prognosis and treatment-related gonadotoxicity. Surgical and radiotherapeutic procedures involving the pelvis or reproductive organs may impair the ability to conceive or carry a pregnancy, while systemic therapies—particularly alkylating agents and conditioning regimens for hematopoietic cell transplantation—are well known to diminish ovarian reserve [19,20]. Among women with breast cancer, chemotherapy combined with prolonged endocrine therapy can delay attempts at pregnancy until later reproductive ages, when fertility is already declining [21]. For CNS tumors, treatment may cause persistent cognitive or physical impairment that further limits the ability or desire to pursue childbearing [22]. Our findings are consistent with these biological mechanisms.

Although information on parity at diagnosis was unavailable in our study, we found age at diagnosis emerged as a major determinant of likelihood of childbirth. Women diagnosed during adolescence or early adulthood had higher cumulative incidences and SBRs, whereas those diagnosed at 35–39 years had sharply reduced measures. This pattern, also observed in other cohorts [17], reflects both age-related declines in ovarian reserve and reduced intention for additional children later in the reproductive life course. Younger survivors may have time to “catch up,” whereas those treated near the end of their reproductive window have limited opportunity to do so. Temporal patterns in our data and studies from other countries [9] suggest modest improvements in reproductive outcomes in more recent treatment eras, potentially due to improved cancer prognosis, greater use of fertility-sparing procedures, and increased access to fertility preservation.

Non-biologic and psychosocial factors also influence childbearing after cancer. Many survivors defer or avoid pregnancy because of fear of recurrence, concerns about heritability or pregnancy complications, financial pressure, or relationship instability [23]. Survivors with prior children may feel their families are complete or may be less willing to assume further risk, whereas nulliparous survivors may be highly motivated to pursue pregnancy, including through assisted reproduction. International data further show that survivors require fertility treatment more frequently than their peers but do not necessarily access fertility services at higher rates [24]. A Texas study found racial and ethnic disparities in livebirth after cancer, with Black survivors and Asian or Pacific Islander survivors experiencing substantially lower rates [25]. In Taiwan, universal health coverage may reduce some financial barriers for cancer care; however, fertility preservation and assisted reproductive technologies were historically less accessible due to high costs, and our study lacked data on use of these services.

These findings have important implications for clinical care and policy. Reduced fertility should be recognized as a common late effect of cancer in young women. Fertility counseling should be integrated into routine care for all reproductive-aged patients, with particular attention to those receiving gonadotoxic treatments or diagnosed at older ages. When clinically appropriate, fertility-sparing surgery and fertility preservation—such as oocyte or embryo cryopreservation—should be discussed before treatment initiation. Establishing clear pathways for timely referral to reproductive specialists, along with survivorship care that revisits reproductive goals and addresses treatment-related ovarian insufficiency, is essential to high-quality oncofertility care [7]. Recently, the Taiwanese government introduced a subsidy program enabling young adults with breast and hematologic cancers to access fertility preservation services, helping to reduce financial barriers. Our nationwide findings may inform policymakers in evaluating whether eligibility for fertility preservation subsidies should be expanded to include survivors of other cancer types with compromised likelihood of childbirth. Furthermore, to address the unmet reproductive needs of survivors with absolute uterine factor infertility, policymakers could initiate evidence-informed discussions on medically indicated gestational surrogacy while also strengthening institutional support for adoption.

Strengths of this study include its nationwide, population-based design, large sample size, and near-complete linkage across cancer, birth, and death registries, allowing robust estimation of cumulative incidence and SBRs across cancer types, ages, and treatment categories. The SBR approach also accounts for secular changes in reproductive behavior in Taiwan. Nonetheless, limitations exist. We lacked information on parity at diagnosis, marital status, socioeconomic factors, fertility intentions, and use of fertility preservation or assisted reproductive technologies, limiting the ability to differentiate biological infertility from voluntary reproductive choices. Treatment information was available only in broad categories, without detailed data on specific agents, doses, or radiation fields. Women who undergo more aggressive treatments are inherently more likely to have been diagnosed with more advanced, high-stage, or biologically aggressive malignancies. Consequently, their lower post-diagnostic childbirth rates may be influenced not only by chemical toxicity but also by the overarching clinical severity of their disease. We were also unable to assess cancer recurrence, pregnancies ending before livebirth, or births occurring outside Taiwan. Finally, women diagnosed in the most recent calendar periods had shorter follow-up, likely underestimating their eventual likelihood of childbirth.

In conclusion, women diagnosed with cancer at age ≤ 39 years in Taiwan experience substantially fewer livebirths than expected, with the greatest deficits among survivors of leukemia, CNS, breast, and gynecological cancers and among those diagnosed at older reproductive ages. Together with international evidence, our findings underscore the combined contributions of treatment-related gonadotoxicity, age at diagnosis, and non-biologic factors to reduced likelihood of childbirth after cancer. Systematic oncofertility counseling, access to fertility preservation and reproductive care, and research incorporating detailed clinical, treatment, and psychosocial data are needed to support informed decision-making and improve reproductive outcomes in this growing population of survivors.

## Supporting information

S1 File PLOS Human Participants Research Checklist 2025. (PDF)
