# Supplementary material for: Rates of childbirth in female cancer survivors: A population-based study in Taiwan
Source: PLoS One. 2026 Sep 11;21(9):e0358466. doi: 10.1371/journal.pone.0358466 (PMC13568492; doi:10.1371/journal.pone.0358466)
Supplement: Peer review history

# Peer review: Decision Letter 0

**DOI:** 10.1371/journal.pone.0358466.r001

**Authors:** Chong-Chi Chiu

16 Jul 2026

Dear Dr. Yu,

Please submit your revised manuscript by Aug 30 2026 11:59PM. If you will need more time than this to complete your revisions, please reply to this message or contact the journal office at plosone@plos.org. When you're ready to submit your revision, log on to https://www.editorialmanager.com/pone/ and select the 'Submissions Needing Revision' folder to locate your manuscript file.

- A letter that responds to each point raised by the academic editor and reviewer(s). You should upload this letter as a separate file labeled 'Response to Reviewers'.
- A marked-up copy of your manuscript that highlights changes made to the original version. You should upload this as a separate file labeled 'Revised Manuscript with Track Changes'.
- An unmarked version of your revised paper without tracked changes. You should upload this as a separate file labeled 'Manuscript'.

If you would like to make changes to your financial disclosure, please include your updated statement in your cover letter. Guidelines for resubmitting your figure files are available below the reviewer comments at the end of this letter.

If applicable, we recommend that you deposit your laboratory protocols in protocols.io to enhance the reproducibility of your results. Protocols.io assigns your protocol its own identifier (DOI) so that it can be cited independently in the future. For instructions see: https://journals.plos.org/plosone/s/submission-guidelines#loc-laboratory-protocols. Additionally, PLOS ONE offers an option for publishing peer-reviewed Lab Protocol articles, which describe protocols hosted on protocols.io. Read more information on sharing protocols at https://plos.org/protocols?utm_medium=editorial-email&utm_source=authorletters&utm_campaign=protocols.

As the corresponding author, your ORCID iD is verified in the submission system and will appear in the published article. PLOS supports the use of ORCID, and we encourage all coauthors to register for an ORCID iD and use it as well. Please encourage your coauthors to verify their ORCID iD within the submission system before final acceptance, as unverified ORCID iDs will not appear in the published article. *Only*  the individual author can complete the verification step; PLOS staff *cannot*  verify ORCID iDs on behalf of authors.

We look forward to receiving your revised manuscript.

Kind regards,

Chong-Chi Chiu

Academic Editor

PLOS One

Journal requirements:

When submitting your revision, we need you to address these additional requirements.

1. Please ensure that your manuscript meets PLOS ONE's style requirements, including those for file naming. The PLOS ONE style templates can be found at

https://journals.plos.org/plosone/s/file?id=wjVg/PLOSOne_formatting_sample_main_body.pdf and

https://journals.plos.org/plosone/s/file?id=ba62/PLOSOne_formatting_sample_title_authors_affiliations.pdf

2. For studies involving third-party data, we encourage authors to share any data specific to their analyses that they can legally distribute. PLOS recognizes, however, that authors may be using third-party data they do not have the rights to share. When third-party data cannot be publicly shared, authors must provide all information necessary for interested researchers to apply to gain access to the data. (https://journals.plos.org/plosone/s/data-availability#loc-acceptable-data-access-restrictions)

For any third-party data that the authors cannot legally distribute, they should include the following information in their Data Availability Statement upon submission:

1) A description of the data set and the third-party source

2) If applicable, verification of permission to use the data set

3) Confirmation of whether the authors received any special privileges in accessing the data that other researchers would not have

4) All necessary contact information others would need to apply to gain access to the data.

3. Your ethics statement should only appear in the Methods section of your manuscript. If your ethics statement is written in any section besides the Methods, please move it to the Methods section and delete it from any other section. Please ensure that your ethics statement is included in your manuscript, as the ethics statement entered into the online submission form will not be published alongside your manuscript.

If the reviewer comments include a recommendation to cite specific previously published works, please review and evaluate these publications to determine whether they are relevant and should be cited. There is no requirement to cite these works unless the editor has indicated otherwise.

Reviewers' comments:

Reviewer's Responses to Questions

**Comments to the Author**

1. Is the manuscript technically sound, and do the data support the conclusions?

Reviewer #1: Partly

Reviewer #2: Yes

Reviewer #3: Yes

Reviewer #4: Yes

**********

2. Has the statistical analysis been performed appropriately and rigorously?

Reviewer #1: Yes

Reviewer #2: Yes

Reviewer #3: Yes

Reviewer #4: Yes

**********

3. Have the authors made all data underlying the findings in their manuscript fully available?

The PLOS Data policy

Reviewer #1: Yes

Reviewer #2: No

Reviewer #3: Yes

Reviewer #4: Yes

**********

4. Is the manuscript presented in an intelligible fashion and written in standard English?

Reviewer #1: Yes

Reviewer #2: Yes

Reviewer #3: Yes

Reviewer #4: Yes

**********

Reviewer #1: 1. On page 6, line 88, the author states: “Cancer treatment was categorized as surgery only; radiation without chemotherapy; chemotherapy; and other.” It is unclear whether these categories are mutually exclusive or if they include combinations. For instance, does "radiation without chemotherapy" also include patients who underwent surgery? Please clarify the specific composition of each treatment group to avoid ambiguity.

2. The authors should provide a clearer rationale for their choice of adjustment variables in the multivariable models. While the models adjusted for cancer type, age at diagnosis, and period of cancer onset, they did not account for the level of urbanization and cancer treatment. This is particularly concerning given that cancer treatment showed a significant association with childbirth in the results. Please justify why these potentially confounding factors were excluded from the final multivariable analysis.

Reviewer #2: This nationwide registry-linkage study from Taiwan assesses childbirth after cancer among women diagnosed at age ≤39 years. Strengths include the use of linked national registries, the large sample size, and the clinical and public health importance of the topic, in addition to being written very clearly and concisely. Overall, with several revisions, this manuscript should be of interest to readers of PLOS One, fills an important gap in the literature, and has clear implications for survivorship care, fertility counseling, and policy.

Major comments

My primary concern is the SBR analysis. This comparison would be substantially stronger if the authors used a matched non-cancer comparison group rather than an indirectly standardized birth ratio. If a matched analysis is not feasible, the authors should justify more clearly why the SBR approach was selected and discuss its limitations, particularly given that it appears to standardize only by age and calendar year.

Please justify the use of cause-specific Cox models rather than a competing-risk regression model for the correlates analysis. A cause-specific Cox model estimates the instantaneous hazard among women who remain event-free, whereas a competing-risk regression more directly models the cumulative incidence of the outcome in the presence of death as a competing event. Because the descriptive analyses treat death as a competing risk, a competing-risk regression would appear more closely aligned with the overall analytic framework.

The manuscript appears to use different outcome definitions across analyses. The competing-risk and Cox analyses focus on first post-diagnosis livebirth, whereas the SBR analysis seems to include all post-diagnosis livebirths. Please clarify in the Methods whether this is correct, provide a rationale for using different estimands, and add descriptive information for all livebirths if those data are retained in the SBR analysis, since descriptive tables currently describes first livebirth only.

The policy discussion could be strengthened. Lines 236–238 note that the current policy provides fertility preservation subsidies for young adults with breast or hematologic malignancies. However, the results identify particularly low likelihood of livebirth among survivors of gynecologic cancers. This finding should be discussed more directly as a potential opportunity to improve or expand current fertility preservation policies.

Minor comments

1. Please justify the exclusion of women with <1 year of follow-up. Most of the main analyses, including the competing-risk and Cox models, already account for follow-up time, so this exclusion does not seem necessary unless it was specifically required for the SBR analysis.

2. Please add 1–2 sentences describing the quality and completeness of the linked registries for readers who may be unfamiliar with these data sources. It would also be helpful to state when the cancer registry, birth registry, and mortality data became sufficiently comprehensive for population-based analyses. It would also help to add the start date at which these registries were generally representative of the full population- for example line 127 shows that only 2.4% were diagnosed in or before 2000. I’m guessing this means the registry wasn’t fully functional or operational or complete until just before 2000.

3. Trend testing is mentioned in line 154; please specify in the Methods how trends were tested.

4. Lines 189–191 and line 208 require citation. In addition, lines 189–191 should acknowledge prior U.S. findings of especially low livebirth after gastrointestinal cancers (e.g., Betts et al. 2024), if relevant.

Reviewer #3: The study is methodologically sound, clearly presented, and the conclusions are well supported by the data. I have only a few minor suggestions.

1. Please briefly acknowledge that the association between chemotherapy and reduced childbirth may reflect both treatment-related gonadotoxicity and confounding by indication, as women receiving chemotherapy are more likely to have advanced or aggressive disease. In addition, because the registry only captures broad treatment categories, the lack of information on specific agents, treatment intensity, and cumulative doses should be acknowledged when interpreting these findings.

2. Please clarify that the lower childbirth rate among women diagnosed at older reproductive ages may reflect both biological aging and pre-existing reproductive choices (e.g., completed family size). Because parity before diagnosis was unavailable, this distinction would help readers interpret the findings appropriately.

3. Please clarify that the lower SBR observed among women diagnosed in the most recent calendar period is likely influenced by the shorter duration of follow-up, and therefore their reproductive outcomes may be underestimated.

Overall, I believe this manuscript is suitable for publication after minor revision.

Reviewer #4: Major Revision

Introduction

1. The description of Taiwan's recently implemented fertility preservation subsidy program would benefit from greater specificity. As fertility preservation methods and subsidy policies vary across countries, the authors should clarify the specific procedures covered by the Taiwanese program (i.e., oocyte cryopreservation for eligible female patients and sperm cryopreservation for eligible male patients) and briefly distinguish this cancer-specific fertility preservation program from the general IVF subsidy available to infertile couples in Taiwan. This clarification is particularly important for international readers and would strengthen the policy context and relevance of the present study.

2. The Introduction could be more focused and lead readers more directly to the central research question. In particular, the discussion of the ASCO fertility preservation guideline does not appear to be closely relevant to the specific objective of this study, which examines livebirth after cancer. The authors may consider removing this portion from the Introduction or, if relevant to the interpretation of the findings, moving it to the Discussion. This would improve the flow and help readers more readily identify the knowledge gap and central research question.

Methods

1. In the first paragraph of the Methods section, the description of the data sources and study period could be clarified. The current paragraph appears to mix the date of data acquisition, the available date ranges of the respective registries, and the actual study inclusion period. Please consider presenting these separately and clearly specify the calendar period during which cancer diagnoses were eligible for inclusion. For example:

September 1, 2025: date on which the researchers obtained the data.

1979–2022: availability period of the Taiwan Cancer Registry.

2001–2023: availability period of the Birth Reporting Registry.

Up to 2023: availability period of the Taiwan Death Registry.

Actual cancer diagnosis inclusion period: currently unclear.

Clearly distinguishing these different time periods would make the study population and follow-up framework easier to understand and improve reproducibility.

2. In the second paragraph, for readers who are not familiar with cancer registry classification systems, the reference to the ICD-O-3/WHO 2008 definitions may appear outdated, particularly given the long study period and substantial changes in cancer classification over time. A brief clarification of how cancer diagnoses across different calendar periods were harmonized would help reassure readers regarding complete case ascertainment and consistent classification. For example, the original sentence could be revised to:

“Cancer types were classified into mutually exclusive categories according to the ICD-O-3/WHO 2008 definitions, with cancer diagnoses across different calendar periods harmonized to this common classification system.”

3. The authors excluded livebirths occurring within one year after cancer diagnosis. A brief justification for this methodological choice, for example by citing the previous study protocol on which this approach was based, would improve clarity and reproducibility.

Results and Discussion

1. The terms “fertility” and “reproductive potential” appear to be used interchangeably with post-diagnosis livebirth throughout the Results and Discussion, although these are not equivalent outcomes. This study directly assessed post-diagnosis livebirth rather than biological fertility, ovarian reserve, pregnancy attempts, or reproductive intentions.

For example, the statement that “fertility was most preserved among survivors of skin and thyroid cancers” may not be fully supported by the data. The higher livebirth rate observed among thyroid cancer survivors could reflect multiple factors, including favorable prognosis, longer survival, less gonadotoxic treatment, age distribution, or reproductive choices, rather than preserved biological fertility alone.

Similarly, the conclusion that the findings “underscore the combined contributions of treatment-related gonadotoxicity, age at diagnosis, and non-biologic factors to reduced fertility after cancer” appears stronger than what can be directly inferred from the study, as neither biological fertility nor gonadotoxicity was directly assessed.

The authors may consider using more precise terms such as “post-diagnosis livebirth,” “childbearing,” or “likelihood of livebirth” throughout the manuscript and revising the conclusion to more closely reflect the measured outcome. For example:

“Together with international evidence, our findings suggest that reduced post-diagnosis childbearing may reflect the combined influence of treatment-related gonadotoxicity, age at diagnosis, and non-biologic factors.”

2. The interpretation of the association between chemotherapy and subsequent childbirth should be more cautious. The manuscript reports that chemotherapy was associated with a 48% reduction in subsequent childbirth compared with surgery alone; however, the reported HR of 0.52 is unadjusted. Patients receiving chemotherapy may differ substantially from those receiving surgery alone with respect to cancer type, age, disease characteristics, prognosis, and other factors. In addition, treatment information was available only in broad categories without details regarding specific agents, cumulative doses, conditioning regimens, or other gonadotoxic exposures. Therefore, the observed association should not be interpreted as directly demonstrating chemotherapy-related gonadotoxicity, and the authors should consider acknowledging the potential for confounding more explicitly.

3. The Discussion could be shortened and made more focused. Several paragraphs provide overlapping explanations for reduced post-diagnosis childbearing, including disease prognosis, treatment-related gonadotoxicity, age-related biological constraints, reproductive intentions, and psychosocial factors. Consolidating these overlapping sections would improve conciseness and allow the principal findings of the study to remain more prominent.

Minor Revision

Introduction

1. The opening sentence, “Cancer is sometimes diagnosed at a young age,” is overly general and does not effectively engage the reader or introduce the central focus of the study. Given that the study examines livebirth after cancer among young females, the authors may consider opening more directly with the growing importance of reproductive outcomes among young cancer survivors in the context of improving cancer survival.

Methods

1. Line 94: The first sentence of this subsection is redundant, as the availability of birth records from 2001 to 2023 has already been described in the Data source section, and may be deleted for conciseness.

**********

what does this mean?). If published, this will include your full peer review and any attached files.

If you choose “no”, your identity will remain anonymous but your review may still be made public.

**Do you want your identity to be public for this peer review?**  For information about this choice, including consent withdrawal, please see our Privacy Policy

Reviewer #1: No

Reviewer #2: No

Reviewer #3: **Yes:** Ya-Yun Cheng

Reviewer #4: No

**********

[NOTE: If reviewer comments were submitted as an attachment file, they will be attached to this email and accessible via the submission site. Please log into your account, locate the manuscript record, and check for the action link "View Attachments". If this link does not appear, there are no attachment files.]

To ensure your figures meet our technical requirements, please review our figure guidelines: https://journals.plos.org/plosone/s/figures

You may also use PLOS’s free figure tool, NAAS, to help you prepare publication quality figures: https://journals.plos.org/plosone/s/figures#loc-tools-for-figure-preparation.

NAAS will assess whether your figures meet our technical requirements by comparing each figure against our figure specifications.

# Author comment: Author response to Decision Letter 1

**DOI:** 10.1371/journal.pone.0358466.r002

23 Jul 2026

Dear editors,

Thank you for reviewing our manuscript. The comments were very helpful for us to revise and improve the manuscript. Hereby please find below point-by-point responses to the editor’s comments. We also attached the revised manuscript with tracked changes and a clean version.

We look forward to seeing your further evaluation and response.

Sincerely yours,

Tsung Yu, PhD

Response to Reviewers

Reviewer #1:

1. On page 6, line 88, the author states: “Cancer treatment was categorized as surgery only; radiation without chemotherapy; chemotherapy; and other.” It is unclear whether these categories are mutually exclusive or if they include combinations. For instance, does "radiation without chemotherapy" also include patients who underwent surgery? Please clarify the specific composition of each treatment group to avoid ambiguity.

Response:

We thank the reviewer for this important comment. We apologize for the ambiguity in our initial description. We would like to clarify that these four cancer treatment categories are mutually exclusive and were constructed hierarchically based on the potential gonadotoxicity of the therapies to avoid overlap. The specific composition and hierarchy of each group are defined as follows:

(1) Chemotherapy: Includes all patients who received chemotherapy, regardless of whether they also underwent surgery or radiation therapy. This was prioritized because systemic chemotherapy generally carries the highest risk of gonadotoxicity.

(2) Radiation, no chemotherapy: Includes patients who received radiation therapy (with or without surgery) but did not receive any chemotherapy.

(3) Surgery only: Includes patients who underwent surgical procedures only, with no subsequent radiation or chemotherapy.

(4) Other: Includes individuals with other treatment modalities, missing data, or unspecified registry records. To improve clarity, we have revised the text in the Methods section (Page 7, Lines 101-103 in the manuscript with tracked changes) as suggested.

2. The authors should provide a clearer rationale for their choice of adjustment variables in the multivariable models. While the models adjusted for cancer type, age at diagnosis, and period of cancer onset, they did not account for the level of urbanization and cancer treatment. This is particularly concerning given that cancer treatment showed a significant association with childbirth in the results. Please justify why these potentially confounding factors were excluded from the final multivariable analysis.

Response:

We thank the reviewer for pointing out this methodological nuance. We would like to provide the epidemiological and clinical rationale for our variable selection in the final multivariable model:

(1) Cancer Treatment: In causal inference, a confounder must cause both the exposure (cancer type) and the outcome (childbirth). In our study, cancer treatment does not cause the cancer type; rather, the specific cancer type and stage dictate the treatment modality (e.g., breast cancer leading to chemotherapy). Therefore, cancer treatment acts as a mediator on the causal pathway between cancer diagnosis and subsequent fertility.

(2) Urbanization Level: Prior studies in Taiwan indicate that while urbanization correlates with healthcare access, Taiwan’s National Health Insurance (NHI) program provides universal coverage, minimizing geographic disparities in major cancer care and fertility consultations. In our univariate analysis, urbanization level did not show a statistically significant independent association with childbirth (p >0.05).

Thereby, we did not include these two factors in the multivariable models. To clarify this rationale for readers, we have added sentences explaining our variable selection and causal framework in the Methods section (Page 8, Lines 124-126).

Reviewer #2:

3. This nationwide registry-linkage study from Taiwan assesses childbirth after cancer among women diagnosed at age ≤39 years. Strengths include the use of linked national registries, the large sample size, and the clinical and public health importance of the topic, in addition to being written very clearly and concisely. Overall, with several revisions, this manuscript should be of interest to readers of PLOS One, fills an important gap in the literature, and has clear implications for survivorship care, fertility counseling, and policy.

Major comments

My primary concern is the SBR analysis. This comparison would be substantially stronger if the authors used a matched non-cancer comparison group rather than an indirectly standardized birth ratio. If a matched analysis is not feasible, the authors should justify more clearly why the SBR approach was selected and discuss its limitations, particularly given that it appears to standardize only by age and calendar year.

Response:

Due to data access limitations within the national registries, individual-level data for a matched non-cancer cohort were not available to our research team. Instead, we utilized aggregate-level annual fertility data of the entire Taiwanese female population. Under these data conditions, the indirect standardization approach (SBR) is the epidemiologically preferred and mathematically robust method for several reasons:

(1) Prevention of Selection Bias: Comparing our cohort directly to the entire general population eliminates the risk of selection bias that could accidentally occur when drawing a smaller, sampled matched control group from the public.

(2) Statistical Stability: The general population provides an extremely stable and unfluctuating baseline for birth rates, which is ideal when evaluating a specific, smaller exposed group (cancer survivors). This approach mirrors the standard method used for calculating Standardized Incidence Ratios (SIR) in cancer registry studies worldwide.

(3) Perfect Temporal Alignment: Standardizing by both age and specific calendar years effectively controls for the significant temporal declines in fertility rates that Taiwan has experienced over the past decades. To address the reviewer's concern and ensure full transparency, we have expanded our explanation justifying the SBR approach and have explicitly acknowledged the reliance on aggregate-level population data in the Methods section (Page 9, Lines 135-139).

4. Please justify the use of cause-specific Cox models rather than a competing-risk regression model for the correlates analysis. A cause-specific Cox model estimates the instantaneous hazard among women who remain event-free, whereas a competing-risk regression more directly models the cumulative incidence of the outcome in the presence of death as a competing event. Because the descriptive analyses treat death as a competing risk, a competing-risk regression would appear more closely aligned with the overall analytic framework.

Response:

We thank the reviewer for this sophisticated methodological comment regarding survival analysis in the presence of competing risks. We agree that choosing between a cause-specific Cox model and a subdistribution hazard model (Fine and Gray method) is a critical decision in competing-risk frameworks. We would like to justify our selection of the cause-specific Cox model based on standard epidemiological guidelines (e.g., Lau et al., Am J Epidemiol, 2009).

The primary objective of our multivariable analysis is to investigate the etiological and biological impacts of cancer types and treatment modalities (e.g., chemotherapy) on subsequent fertility and ovarian reserve. Epidemiological literature establishes that the cause-specific hazard ratio (csHR) is the preferred measure when studying the biological etiology of an exposure on an outcome. This is because it estimates the instantaneous risk among patients who are currently alive and capable of experiencing the event, without being artifactually altered by the mortality rate of the specific cancer type.

5. The manuscript appears to use different outcome definitions across analyses. The competing-risk and Cox analyses focus on first post-diagnosis livebirth, whereas the SBR analysis seems to include all post-diagnosis livebirths. Please clarify in the Methods whether this is correct, provide a rationale for using different estimands, and add descriptive information for all livebirths if those data are retained in the SBR analysis, since descriptive tables currently describes first livebirth only.

Response:

The reviewer is entirely correct, and we appreciate this opportunity to clarify our analytical approach. In our study, the survival analyses (cause-specific Cox models) focus strictly on the first post-diagnosis livebirth, whereas the SBR analysis encompasses all post-diagnosis livebirths contributed by the cohort. The cause-specific Cox models operate under a standard time-to-first-event framework. This allows us to investigate the specific biological and treatment-related factors (e.g., chemotherapy vs. surgery) that influence a survivor's initial capability to achieve parenthood after diagnosis. The SBR analysis captures the total, cumulative reproductive deficit or output of the entire survivor population relative to the general public. By including subsequent births (second or later children), the SBR reflects the comprehensive, long-term societal and demographic impact of cancer on these families' complete family-building process. Furthermore, as suggested by the reviewer, we would like to highlight that the descriptive information regarding all post-diagnosis livebirths (including the total count and total distribution) is provided and summarized in Table 4.

6. The policy discussion could be strengthened. Lines 236–238 note that the current policy provides fertility preservation subsidies for young adults with breast or hematologic malignancies. However, the results identify particularly low likelihood of livebirth among survivors of gynecologic cancers. This finding should be discussed more directly as a potential opportunity to improve or expand current fertility preservation policies.

Response:

We thank the reviewer for this insightful and constructive recommendation. We completely agree that our finding regarding the severely low livebirth rate among gynecologic cancer survivors highlights a critical gap in current healthcare policy. Following the reviewer's suggestion, we have expanded the policy discussion in the Discussion section (Pages 15-16, Lines 270-274).

7. Minor comments

Please justify the exclusion of women with <1 year of follow-up. Most of the main analyses, including the competing-risk and Cox models, already account for follow-up time, so this exclusion does not seem necessary unless it was specifically required for the SBR analysis.

Response:

We thank the reviewer for this critical methodological comment. We appreciate the opportunity to clarify the rationale behind this exclusion criterion, which was applied uniformly across all analyses. The primary justification for excluding women with less than 1 year of follow-up is to ensure that all analyzed livebirths were conceived strictly after the cancer diagnosis, thereby capturing true post-diagnostic fertility. To enhance transparency and justify this methodological choice, we have added this explicit rationale to the Methods section (Page 8, Lines 117-118).

8. Please add 1–2 sentences describing the quality and completeness of the linked registries for readers who may be unfamiliar with these data sources. It would also be helpful to state when the cancer registry, birth registry, and mortality data became sufficiently comprehensive for population-based analyses. It would also help to add the start date at which these registries were generally representative of the full population- for example line 127 shows that only 2.4% were diagnosed in or before 2000. I’m guessing this means the registry wasn’t fully functional or operational or complete until just before 2000.

Response:

We thank the reviewer for this question regarding the infrastructure of the linked national registries. We would like to clarify that the low proportion (2.4%) of patients diagnosed in or before 2000 is not due to the incompleteness of the Taiwan Cancer Registry at that time, but rather due to the temporal availability of our Birth Registry data (which covers the years 2001 to 2023). Because livebirth events could only be captured from 2001 onward, patients diagnosed in or before 2000 were only eligible for inclusion in our study if they were diagnosed at a very young age (e.g., childhood or early adolescence) and survived to potentially achieve a livebirth within our 2001–2023 tracking window. Following your suggestion, we have added sentences clarifying this issue in the Results section (Page 10, Lines 155-158).

9. Trend testing is mentioned in line 154; please specify in the Methods how trends were tested.

Response:

We thank the reviewer for pointing out this omission. We would like to clarify our statistical approach for the trend analysis. To test for linear trends across categorical categories (such as age groups or periods of cancer onset), we assigned sequential integer scores (i.e., 1, 2, 3, 4...) to the median or ordered values of each category. These scores were then entered into the regression models as an ordinal continuous covariate. The statistical significance of the linear trend was evaluated using the Wald test. As requested, we have added this methodological specification to the Methods section (Page 8, Lines 127-129) to ensure complete reproducibility.

10. Lines 189–191 and line 208 require citation. In addition, lines 189–191 should acknowledge prior U.S. findings of especially low livebirth after gastrointestinal cancers (e.g., Betts et al. 2024), if relevant.

Response:

We have now added appropriate, relevant citations to these lines in the revised Discussion section (Page 13, Line 222). Our national data did not, however, show an especially low likelihood of livebirth among survivors of gastrointestinal cancers compared to other cancer types in Taiwan.

Reviewer #3:

11. The study is methodologically sound, clearly presented, and the conclusions are well supported by the data. I have only a few minor suggestions. Please briefly acknowledge that the association between chemotherapy and reduced childbirth may reflect both treatment-related gonadotoxicity and confounding by indication, as women receiving chemotherapy are more likely to have advanced or aggressive disease. In addition, because the registry only captures broad treatment categories, the lack of information on specific agents, treatment intensity, and cumulative doses should be acknowledged when interpreting these findings.

Response:

We agree that these considerations are vital for a comprehensive interpretation of our findings. Following the reviewer’s recommendations, we have carefully revised our Discussion section (Page 16, Lines 282-285).

12. Please clarify that the lower childbirth rate among women diagnosed at older reproductive ages may reflect both biological aging and pre-existing reproductive choices (e.g., completed family size). Because parity before diagnosis was unavailable, this distinction would help readers interpret the findings appropriately.

Response:

We are grateful to the reviewer for highlighting this critical distinction, and we are pleased to note that our clinical reasoning aligns perfectly with the reviewer’s perspective. In our manuscript, we did acknowledge the data constraint regarding the lack of baseline parity information on Page 14, Lines 240-245.

13. Please clarify that the lower SBR observed among women diagnosed in the most recent calendar period is likely influenced by the shorter duration of follow-up, and therefore their reproductive outcomes may be underestimated.

Overall, I believe this manuscript is suitable for publication after minor revision.

Response:

As noted by the reviewer, the shorter duration of follow-up for the most recently diagnosed cohort is indeed a critical factor that could temporarily underestimate their long-term reproductive outcomes. We did acknowledge this constraint in our Limitations section on Page 16, Lines 286-288.

Reviewer #4:

14. Major Revision

Introduction

The description of Taiwan's recently implemented f

Attachment Submitted filename: Response to Reviewers.docx

# Peer review: Decision Letter 1

**DOI:** 10.1371/journal.pone.0358466.r003

**Authors:** Chong-Chi Chiu

2 Sep 2026

Rates of childbirth in female cancer survivors: a population-based study in Taiwan

PONE-D-26-17199R1

Dear Dr. Yu,

We’re pleased to inform you that your manuscript has been judged scientifically suitable for publication and will be formally accepted for publication once it meets all outstanding technical requirements.

Within one week, you’ll receive an e-mail detailing the required amendments. When these have been addressed, you’ll receive a formal acceptance letter and your manuscript will be scheduled for publication.

An invoice will be generated when your article is formally accepted. Please note, if your institution has a publishing partnership with PLOS and your article meets the relevant criteria, all or part of your publication costs will be covered. Please make sure your user information is up-to-date by logging into Editorial Manager at Editorial Manager® and clicking the ‘Update My Information' link at the top of the page. For questions related to billing, please contact billing support.

If your institution or institutions have a press office, please notify them about your upcoming paper to help maximize its impact. If they’ll be preparing press materials, please inform our press team as soon as possible -- no later than 48 hours after receiving the formal acceptance. Your manuscript will remain under strict press embargo until 2 pm Eastern Time on the date of publication. For more information, please contact onepress@plos.org.

Kind regards,

Chong-Chi Chiu

Academic Editor

PLOS One

Reviewers' comments:

Reviewer's Responses to Questions

**Comments to the Author**

Reviewer #1: All comments have been addressed

Reviewer #2: All comments have been addressed

Reviewer #3: All comments have been addressed

**********

2. Is the manuscript technically sound, and do the data support the conclusions?

Reviewer #1: Yes

Reviewer #2: Yes

Reviewer #3: Yes

**********

3. Has the statistical analysis been performed appropriately and rigorously?

Reviewer #1: Yes

Reviewer #2: Yes

Reviewer #3: Yes

**********

4. Have the authors made all data underlying the findings in their manuscript fully available?

The PLOS Data policy

Reviewer #1: Yes

Reviewer #2: No

Reviewer #3: Yes

**********

5. Is the manuscript presented in an intelligible fashion and written in standard English?

Reviewer #1: Yes

Reviewer #2: Yes

Reviewer #3: Yes

**********

Reviewer #1: The authors have adequately addressed my comments raised in a previous round of review and this manuscript is now acceptable for publication.

Reviewer #2: extremely minor- but one response to the review created a little confusion for me. The manuscript mentions testing for ordinal trends across age and period of cancer onset (127-129) but only one trend test is presented (by time); they did not include p-trend for age results in the paper. If they had they done test for trend by age group, using the median of each group would have been more appropriate than using sequential integers. I think they can likely delete mention of age group in that area of the manuscript, and then it would be ready for acceptance.

Reviewer #3: The authors have adequately addressed all of my previous comments, particularly regarding the interpretation of chemotherapy-related findings, reproductive outcomes among women diagnosed at older ages, and the shorter follow-up duration in the most recent diagnostic cohort. The revised manuscript is clearer and appropriately acknowledges these limitations. I have no further comments and support publication.

**********

what does this mean?). If published, this will include your full peer review and any attached files.

If you choose “no”, your identity will remain anonymous but your review may still be made public.

**Do you want your identity to be public for this peer review?**  For information about this choice, including consent withdrawal, please see our Privacy Policy

Reviewer #1: No

Reviewer #2: No

Reviewer #3: **Yes:** Ya-Yun Cheng

**********

# Editor report: Acceptance letter

**DOI:** 10.1371/journal.pone.0358466.r004

**Authors:** Chong-Chi Chiu

PONE-D-26-17199R1

PLOS One

Dear Dr. Yu,

I'm pleased to inform you that your manuscript has been deemed suitable for publication in PLOS One. Congratulations! Your manuscript is now being handed over to our production team.

At this stage, our production department will prepare your paper for publication. This includes ensuring the following:

* All references, tables, and figures are properly cited

* All relevant supporting information is included in the manuscript submission,

* There are no issues that prevent the paper from being properly typeset

You will receive further instructions from the production team, including instructions on how to review your proof when it is ready. Please keep in mind that we are working through a large volume of accepted articles, so please give us a few days to review your paper and let you know the next and final steps.

Lastly, if your institution or institutions have a press office, please let them know about your upcoming paper now to help maximize its impact. If they'll be preparing press materials, please inform our press team within the next 48 hours. Your manuscript will remain under strict press embargo until 2 pm Eastern Time on the date of publication. For more information, please contact onepress@plos.org.

You will receive an invoice from PLOS for your publication fee after your manuscript has reached the completed accept phase. If you receive an email requesting payment before acceptance or for any other service, this may be a phishing scheme. Learn how to identify phishing emails and protect your accounts at https://explore.plos.org/phishing.

If we can help with anything else, please email us at customercare@plos.org.

Thank you for submitting your work to PLOS One and supporting open access.

Kind regards,

PLOS One Editorial Office Staff

on behalf of

Professor Chong-Chi Chiu

Academic Editor

PLOS One
